# A Case of a Mass of the Pancreatic Head Presenting as Mixed Hemorrhagic and Septic Shock

**DOI:** 10.7759/cureus.32682

**Published:** 2022-12-19

**Authors:** Ange Ahoussougbemey Mele, Nikhilesh Thapa, Celine A Fadel, Oluseyi Abidoye, Alison Moe, Daniel Castresana

**Affiliations:** 1 Internal Medicine, Northeast Georgia Medical Center Gainesville, Gainesville, USA; 2 Internal Medicine, Northeast Georgia Medical Center Gainsville, Gainesville, USA; 3 Gastroenterology, Northeast Georgia Medical Center Gainesville, Gainesville, USA

**Keywords:** antibiotics therapy, endoscopic retrograde cholangiopancreatography (ercp), pancreatoduodenectomy, pancreatic head adenocarcinoma, acute cholangitis

## Abstract

Acute cholangitis is a biliary tract infection secondary to the obstruction, which causes biliary stasis and bacterial overgrowth. Typically, it presents with the Charcot triad of right upper quadrant abdominal pain, jaundice, and fever. Most acute cholangitis cases are secondary to choledocholithiasis. There are rare cases resulting from pancreatic neoplasm. We report the case of a 43-year-old Caucasian male who was found unresponsive at home with hypotension, anemia, and severe jaundice. Initial imaging studies were notable for a periampullary mass lesion causing intrahepatic biliary ductal dilation. Endoscopic retrograde cholangiopancreatography (ERCP) revealed an actively oozing periampullary fungating mass. In this case, acute cholangitis and hemorrhagic shock secondary to bleeding periampullary lesions are atypical. This case presents an effective treatment plan for this condition.

## Introduction

Acute cholangitis can be the initial presentation of pancreatic head adenocarcinoma [1]. When patients present with acute cholangitis, it is crucial to promptly identify the underlying pathology through endoscopic retrograde cholangiopancreatography (ERCP) and effectively treat it [1]. Treatment involves prompt initiation of antibiotics and pressure support when needed [1]. In the following lines, we discuss the management strategies of a case of hemorrhagic shock and acute cholangitis secondary to pancreatic head adenocarcinoma with duodenal invasion. 

## Case presentation

A 43-year-old Caucasian male with a past medical history of hepatitis A virus infection and obesity was brought to the Emergency Department after being unresponsive at home. Upon initial examination, he was notably obtunded and hypotensive with a blood pressure of 83/22 mmHg. His venous blood gas was notable for respiratory acidosis with a pH of 6.834 and a partial pressure of carbon dioxide (pCO2) of 68.9, and he underwent intubation for airway protection. He received 2 liters of lactated Ringer's bolus and continuous lactated Ringer's infusion but remained hypotensive. Due to persistent shock, he was started on norepinephrine and then vasopressin. 

Laboratory studies were significant for bicarbonate of 12 mmol/L, and total bilirubin of >20 mg/dL with a direct bilirubin of 14.4 mg/dL. The patient had marked elevation in aspartate aminotransferase of 284 U/L and alanine aminotransferase of 188 U/L. His creatinine was 1.29 mg/dL and international normalized ratio (INR) was >10. Complete blood count showed a hemoglobin level of 4.2 g/dL, hematocrit of 14.8 %, and a white blood cell count of 75.1 K/uL. Creatine kinase was 3360 U/L and high-sensitivity troponin was 54 ng/L. Other remarkable abnormalities were ammonia of 257 umol/L, elevated lactate dehydrogenase at 536 U/L, and uric acid of 10.3 mg/dL (Table 1). Two blood cultures were negative, and the methicillin-resistant and sensitive *Staphylococcus aureus *(MRSA/MSSA) screens were also negative. His respiratory culture was positive for a heavy growth of *Citrobacter koseri*. He had a negative drug screen. An unclear source of bleeding resulted in acute liver failure. Therefore, the patient received octreotide and pantoprazole bolus and infusions. He received four units of packed red blood cells (PRBCs) and two units of fresh frozen plasma (FFP). He received empiric antibiotics with intravenous meropenem and vancomycin. The initial presentation was suspicious for acute liver failure (ALF).

**Table 1:** Summary of laboratory findings PCO2: partial pressure of carbon dioxide

Laboratory tests	Values
Hemoglobin	4.2 g/dL
Hematocrit	14.8%
White blood cell count	75.1 K/uL
Platelet count	727 k/uL
Sodium	131 mmol/L
Potassium	5.1 mmol/L
Chloride	96 mmol/L
bicarbonate	12 mmol/L
Calcium	8 mg/dL
Protime	>120 seconds
Activated Partial Thromboplastin Time	92.6 seconds
International Normalized Ratio	>10 INR
Troponins	54 ng/L
Electrocardiogram (initial)	Normal sinus rhythm with a rate of 100
Creatinine	1.29 mg/dL
Total bilirubin	>20 mg/dL
Direct bilirubin	14.4 mg/dL
Aspartate aminotransferase	284 U/L
Alanine Aminotransferase	188 U/L
Creatine kinase	3360 U/L
Lactate dehydrogenase	536 U/L
Uric acid	10.3 mg/dL
pH venous blood gas	6.834
PCO2 venous blood gas	68.9
Respiratory culture	Citrobacter Koseri

**Figure 1:**
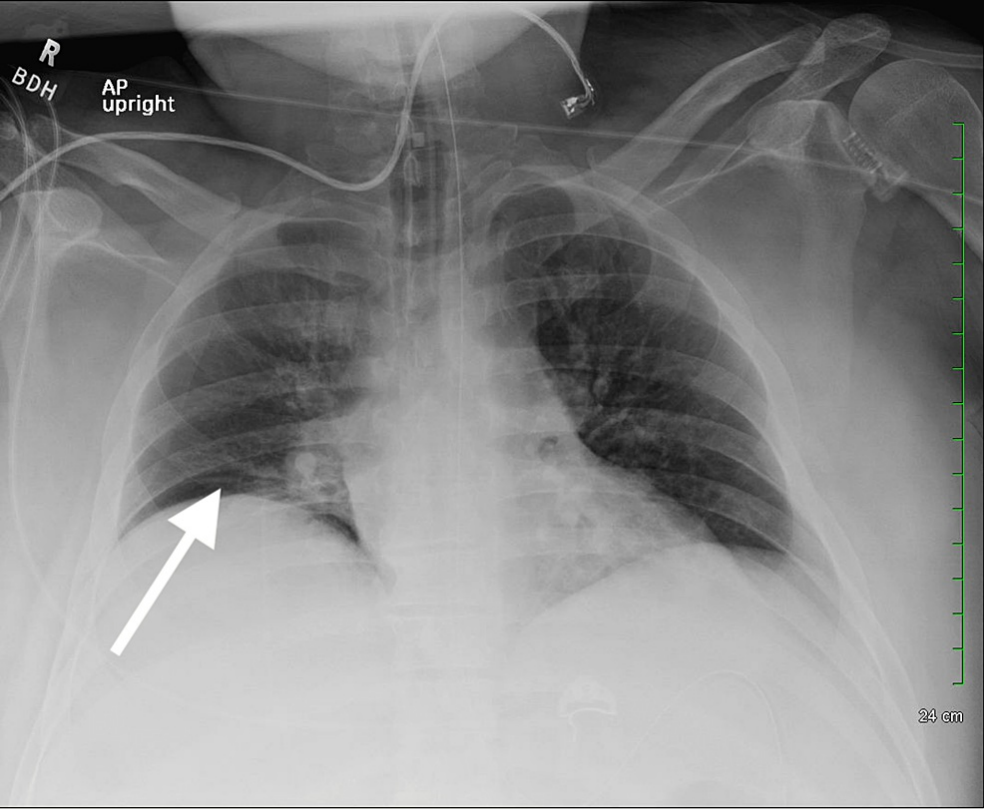
Chest X-ray showing the right basilar sub-segmental atelectasis (arrow)

**Figure 2:**
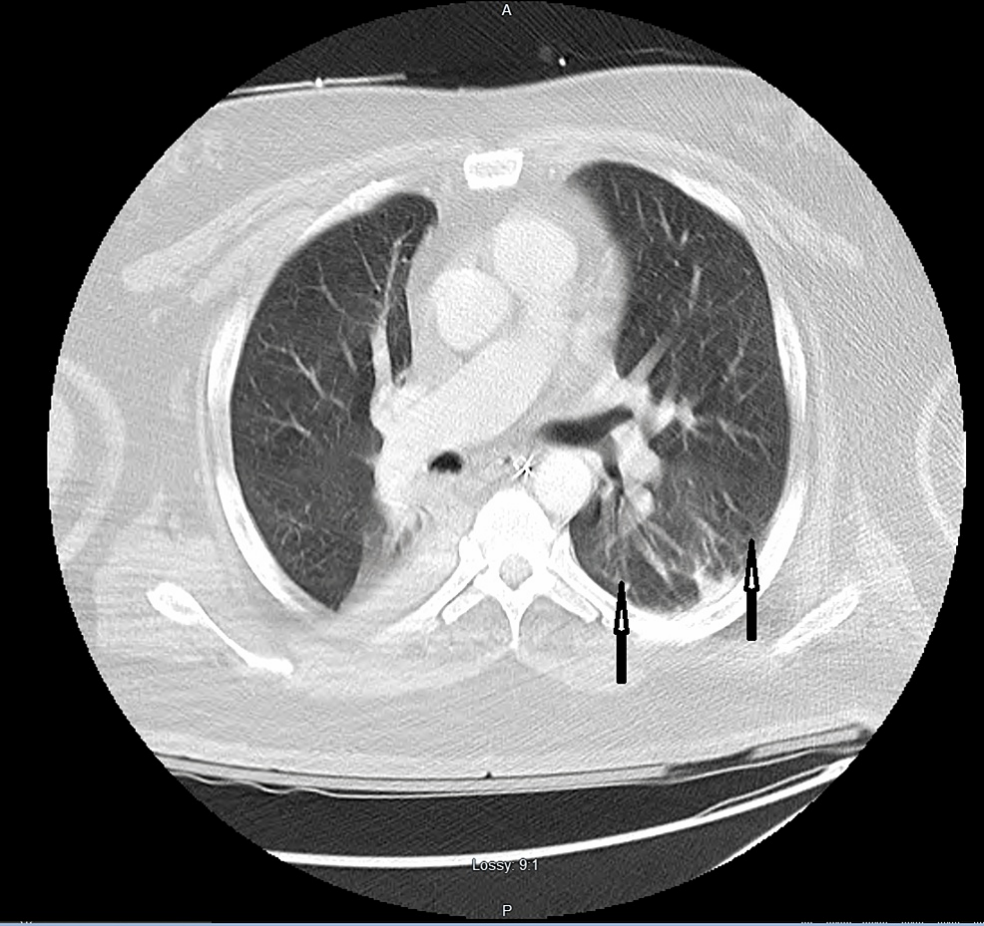
CTA pulmonary showing dependent atelectasis and airspace disease (arrows) CTA: computed tomography angiography

**Figure 3:**
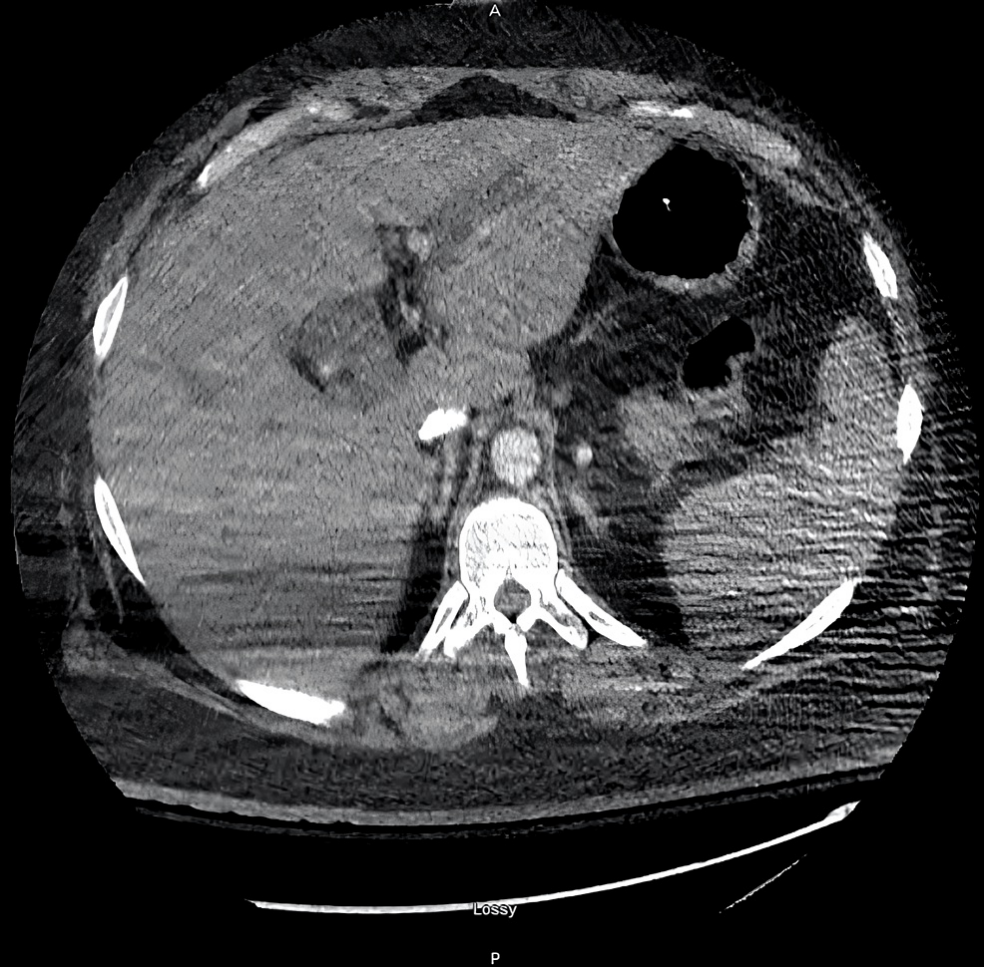
CTA pulmonary showing hepatosplenomegaly CTA: computed tomography angiography

**Figure 4:**
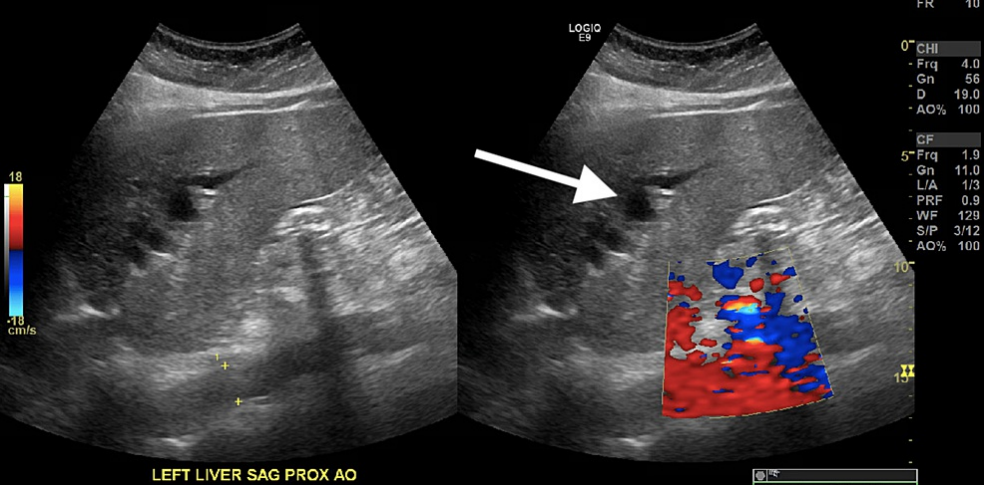
Ultrasound of the right-upper quadrant showing dilation of the bile ducts (arrow), which appear black in contrast to the blood vessels, which have a doppler signal

Imaging studies were performed, which revealed right basilar subsegmental atelectasis on chest radiography (Figure 1). Computed Tomography (CT) brain was negative for any acute abnormality. Pulmonary Computed Tomography Angiography (CTA) was negative for pulmonary embolism. It, however, showed bilateral dependent atelectasis, airspace disease, and hepatosplenomegaly with fatty liver (Figures 2, 3). Right upper quadrant ultrasound was notable for marked prominence of intrahepatic and extrahepatic biliary ducts. In addition, the common bile duct measured up to 2 cm, and there was dilation of the pancreatic duct (Figure 4). 

**Figure 5:**
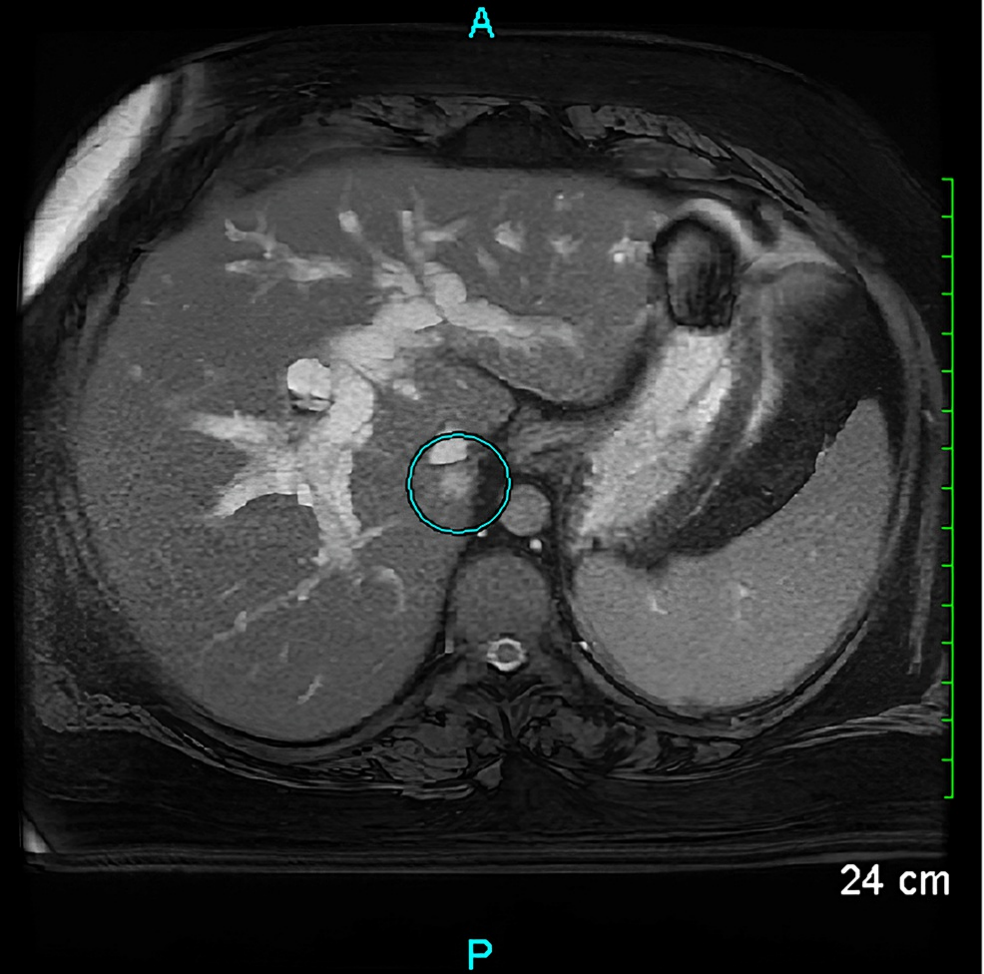
Magnetic resonance imaging of the abdomen with contrast showing heterogenous irregular mass lesion at the periampullary region measuring approximately 2.5 cm

Magnetic resonance cholangiopancreatography (MRCP) showed dilated common bile duct with tapering at the level of the head of the pancreas, suggesting a possible pancreatic head mass or mass at the junction of the common bile duct and duodenum. MRI of the abdomen showed a heterogenous and irregular mass lesion at the periampullary region. This lesion measures approximately 2.5 cm (Figure 5). 

**Figure 6:**
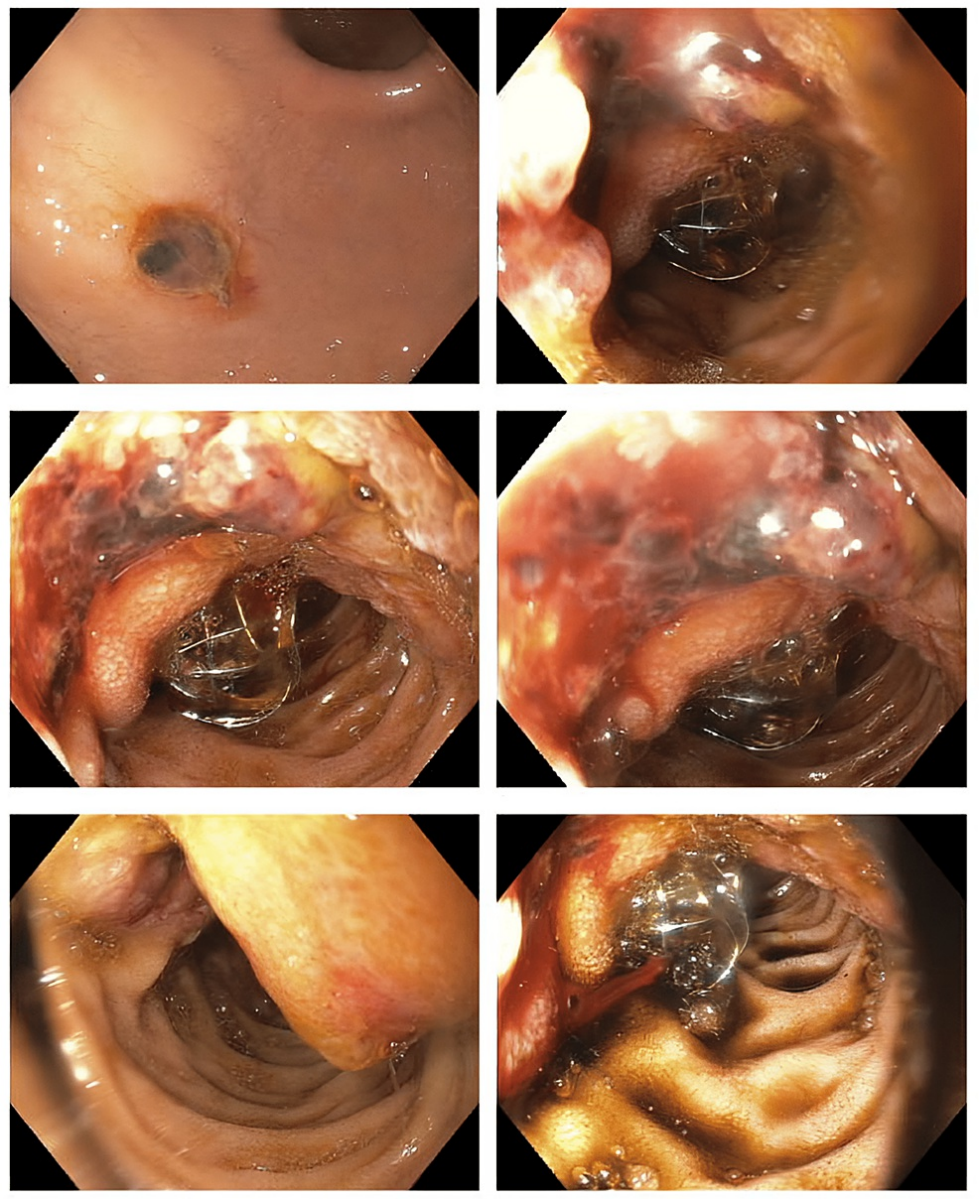
Endoscopic retrograde cholangiopancreatography showing malignant infected distal biliary obstruction status post biliary sphincterotomy, brushings, bile aspiration and placement of 10 mm x 40 mm metal biliary stent. Malignant-appearing periampullary fungating, ulcerative mass.

**Figure 7:**
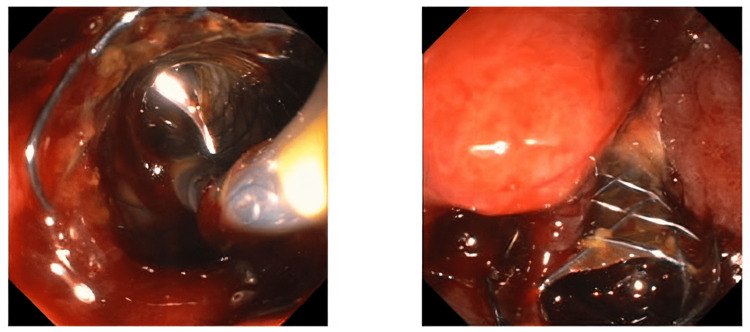
Impending malignant gastric outlet obstruction, status post placement of an additional uncovered metal biliary stent to prevent future biliary obstruction from tumor progression.

The patient underwent urgent ERCP, given findings of acute cholangitis with biliary obstruction and severe septic shock. The ERCP revealed a malignant, infected distal biliary obstruction secondary to a periampullary mass. During ERCP, the patient underwent sphincterotomy, brushings, bile aspiration for culture, and placement of a 10 mm x 40 mm metal biliary stent (Figures 6, 7). Gross inspection revealed a malignant appearing periampullary fungating, ulcerative mass that we biopsied. Bile aspiration did not grow any organisms. 

Post-ERCP, the patient was noted to have significant hematochezia, which was not present before ERCP. Given the friable oozing mass noted during ERCP, the patient underwent esophagogastroduodenoscopy. It revealed a large, ulcerated mass in the periampullary region with active oozing noted. Metal biliary stent remained in place. Multiple applications of hemospray allowed to achieve hemostasis. The patient received pantoprazole bolus and infusion. He remained hemodynamically stable with no further signs of bleeding. 

Postoperatively, his clinical course continued to improve. His antibiotics upon admission were IV meropenem and vancomycin and de-escalated to IV levofloxacin and metronidazole before being discontinued. As the patient improved clinically, we stopped norepinephrine and vasopressin.

Based on endoscopic findings, the patient underwent cancer marker testing. Carcinoembryonic antigen (CEA) level resulted at 1.3 ng/ml, and cancer antigen 19-9 (CA 19-9) was elevated at 1716.07 u/mL. The culture of bile aspirate was negative. Pathology results from intraoperative biopsy were consistent with at least intramucosal adenocarcinoma involving the duodenum. Elevated CA 19-9 and average CEA level point toward primary pancreatic adenocarcinoma that has spread to the duodenum. The results effectively ruled out a duodenal adenocarcinoma. 

Approximately seven days post emergent ERCP, the patient was hemodynamically stable. Sepsis secondary to cholangitis from biliary obstruction due to periampullary malignancy was resolved. At the time of discharge, his hemoglobin had improved to 12.6 g/dL, and the total bilirubin was 7 mg/dl. He was discharged home in stable condition with plans for repeat ERCP with stent exchange within two weeks.

The patient was also referred to medical oncology and began neoadjuvant chemotherapy with modified FOLFIRINOX (folinic acid, fluorouracil, irinotecan hydrochloride, and oxaliplatin).

## Discussion

Acute cholangitis is an infection of the biliary tract secondary to obstruction. It results in biliary stasis and bacterial overgrowth. The condition typically presents with Charcot triad of right upper quadrant pain, jaundice, and fever. Reynold's pentad occurs when symptoms are associated with septic shock and altered mental status. The initial diagnostic test is a right upper quadrant ultrasound imaging. The laboratory findings in this patient included hyperbilirubinemia, leukocytosis, and elevation in transaminase levels. Endoscopic retrograde cholangiopancreatography is the definitive treatment to evaluate and address the underlying pathology. Treatment also consists of both intravenous fluid administration and antibiotics [1]. 

As reported by Qureshi, empiric antibiotics should cover gram-negative and anaerobic organisms. The initial choice recommended is piperacillin-tazobactam, ticarcillin-clavulanate, ceftriaxone plus metronidazole, or ampicillin-sulbactam. When the patient is sensitive to penicillin derivatives, ciprofloxacin plus metronidazole, carbapenems, or gentamicin plus metronidazole are adequate alternatives [2]. Treatment consists of 7 to 10 days of antibiotics. Tanaka et al., however, relay that only 21% to 71% of cases of acute cholangitis have a positive blood culture [3]. High biliary intraductal pressure impairs biliary secretion of antibiotics. As such, biliary drainage is crucial in managing acute cholangitis. Biliary drainage consists of therapeutic and diagnostic endoscopic retrograde cholangiopancreatography. During this procedure, our patient underwent sphincterotomy, brushings, bile aspiration for culture, and placement of a 10 mm x 40 mm metal biliary stent. Donelli et al. explain that if there is blockage of the existing stent due to the growth of bacterial biofilm and the formation of bile sludge, the clinician should remove the old stent and place a new one [4]. Endoscopic nasobiliary drainage by nasobiliary catheter, percutaneous transhepatic biliary drainage, endoscopic-ultrasound-guided drainage, and open surgical drainage are other ways of completing biliary drainage [4]. The latter method is reserved when other modalities are contraindicated or futile [4]. 

Wada et al. discuss the Tokyo Guidelines for the diagnosis and severity assessment of acute cholangitis. The clinician can classify the severity of acute cholangitis into three grades: mild (grade I), moderate (grade II), and severe (grade III), based on the onset of organ dysfunction and the response to the initial medical treatment [5]. Per the Tokyo Guidelines, severe acute cholangitis (grade III) presents with at least one new-onset organ dysfunction. Moderate acute cholangitis (grade II) occurs without organ dysfunction but does not respond to the initial medical treatment. In this case, the clinical manifestations and laboratory data still need to be improved. Mild (grade I) acute cholangitis responds to the initial medical treatment with improved clinical findings [5]. It is essential to classify acute cholangitis as "severe" or "non-severe" at the time of diagnosis. Patients with severe acute cholangitis require urgent biliary drainage and general and organ-supportive treatment. The clinician should monitor patients with non-severe acute cholangitis to determine whether they respond to the initial medical treatment [5]. 

Per Zhang et al., the prognosis of acute cholangitis depends on the timing of biliary drainage, administration of antibiotics, and patient comorbidities. Early biliary drainage is associated with decreased mortality, and the overall mortality after the completion of biliary drainage is less than 10% [6]. Buyukasik et al. postulate that old age, fever, leukocytosis, hyperbilirubinemia, and hypoalbuminemia are associated with poor prognosis. So are comorbidities such as cirrhosis, malignancy, liver abscess, and coagulopathy [7]. 

For this patient, biopsy results from the periampullary lesion showed intramucosal adenocarcinoma involving the duodenum. The patient's CA 19-9 was high, and CEA was normal. These findings are consistent with primary pancreatic adenocarcinoma rather than duodenal adenocarcinoma. According to Dolay et al., pancreatic head adenocarcinoma typically presents with regional or distant lymph node metastases. Other challenging characteristics of their management are pancreatic and retroperitoneal tissues with positive resection margins [8]. Per Butchler et al., pancreatic head adenocarcinoma spreads early along neural sheaths. As such, only 30% to 40% of surgical removal of the pancreas achieves a negative microscopic margin in which no gross or microscopic tumor remains [9].

The patient will undergo robotic-assisted pancreatoduodenectomy three weeks after the completion of chemotherapy. Kostov et al. state that pancreatectomy is associated with a mortality rate of 0.7-3% and a morbidity rate of 36% to 41% [10]. Per McPhee et al., surgical resection is associated with a five-year overall survival rate of 25% and represents the only chance for a cure [11]. The patient is treated with modified FOLFIRINOX by medical oncology. Per Conroy et al., adjuvant therapy with modified FOLFIRINOX results in a more prolonged survival than gemcitabine in patients with resected pancreatic cancer [12]. 

## Conclusions

In conclusion, acute cholangitis may present with right upper quadrant pain, jaundice, fever, septic shock, and altered mental status. A primary pancreatic head tumor caused it with duodenal involvement in our patient. The prognosis depends on how soon the clinician completes biliary drainage and the patient's comorbidities. Pancreatoduodenectomy remains the main course of treatment. Moreover, treatment with modified FOLFIRINOX is associated with more prolonged survival in patients with resected pancreatic cancer.
